# A complementary feeding and play intervention improves the home environment and mental development among toddlers in rural India

**DOI:** 10.1111/mcn.13066

**Published:** 2020-12-21

**Authors:** Sylvia Fernandez Rao, Margaret E. Bentley, Nagalla Balakrishna, Paula Griffiths, Hilary Creed‐Kanashiro, Shahnaz Vazir, Susan L. Johnson

**Affiliations:** ^1^ Behavioral Science Unit, Extension and Training Division National Institute of Nutrition (ICMR) Hyderabad India; ^2^ Department of Nutrition and Carolina Population Center University of North Carolina at Chapel Hill Chapel Hill North Carolina USA; ^3^ Division of Statistics National Institute of Nutrition (ICMR) Hyderabad India; ^4^ School of Sport, Exercise and Health Sciences Loughborough University Loughborough UK; ^5^ Department of Nutrition Research Instituto de Investigación Nutricional Lima Peru; ^6^ Children's Eating Laboratory, Department of Pediatrics, Section of Nutrition University of Colorado Anschutz Medical Campus Aurora Colorado USA

**Keywords:** behaviour change communication, cognitive development, home visits, integrated intervention, responsive feeding

## Abstract

A cluster randomized trial design was used to test the efficacy of a behaviour change communication intervention on the quality of the home environment and infant development at 15 months of age. Children (*n* = 600) in rural South India were followed from 3 through 15 months of age. The control group (C group) received the standard of care, the complementary feeding group (CF group) received recommendations on complementary foods and the responsive complementary feeding and play group (RCF&P group) received recommendations on complementary foods plus skills on responsive feeding and play. The intervention was delivered in biweekly home visits to caregivers using flip charts. At postintervention, infants (*n* = 521) were assessed for development (Bayley‐II scales) and their home environment was assessed (Home Observation for Measurement of the Environment [HOME] scale). Cluster adjusted analysis of variance showed no significant differences at baseline. The HOME score at 15 months differed by group, *F*(2, 38) = 6.41, *P* = 0.004; the CF and RCF&P groups had higher scores than the C group. Scores on subscales ‘Opportunities for Variety in Daily Stimulation’ and ‘Caregiver Promotion of Child Development’ (CPCD) were higher for the RCF&P group than for the C and CF groups. Mental development index (MDI) scores differed by group, *F*(2, 37) = 3.31, *P* = 0.04, with the RCF&P group showing higher scores than the C group (*P* < 0.04); no differences were noted in psychomotor development index (PDI) scores (*P* = 0.48). The subscales of HOME associated with MDI at 15 months were ‘CPCD’ and ‘Cleanliness of Child’ (*R* ^2^ = 0.076). ‘CPCD’ was also associated with PDI (*R* ^2^ = 0.039). A responsive complementary feeding and play intervention delivered through home visits benefitted children's mental development and caregiving environment at 15 months.

**Key messages**

- Behaviour change communication for integrated nutrition and early child development based on formative research and delivered effectively through home visits can change behaviours that can improve the home environment and promote child development.
- Caregivers' promotion of child development activities and the cleanliness of the child are associated with cognitive development.
- Community mothers with a high school education can deliver BCC on complementary feeding, play and responsive feeding if they are trained and supervised.

## 1 INTRODUCTION

Over 200 million children in low‐ and middle‐income countries do not reach their developmental potential because of poverty, morbidity, malnutrition, poor environmental conditions, (UNICEF, 2016) and lack of developmentally appropriate stimulation at home (Bradley & Corwyn, 2005; Grantham‐McGregor et al., 2007; McCoy et al., 2016; Walker et al., 2007). Responsive and high‐quality interactions during the first year of life have resulted in subsequent improvements in cognitive and linguistic competence among children and also a more secure attachment to their primary caregiver (Bakermans‐Kranenburg, Van Ijzendoorn, & Bradley, 2005). Timely responsiveness to children's cues of satiety and hunger (Black & Aboud, 2011) and active caregiver engagement are critical to a child's development (Bornstein, Tamis‐Lemonda, Hahn, & Haynes, 2008). These skills are important for child feeding and are associated with higher food and nutrient intakes (Bentley, Wasser, & &Creed‐Kanashiro, H.M., 2011). Recommendations for nutrition programmes have focused on encouraging feeding responsivity, particularly during the period of initiation of complementary feeding (PAHO, 2003).

Behaviour change communication (BCC) in intervention trials has resulted in improved infant nutrition (Hoddinott, Ahmed, Ahmed, & Roy, 2017; Penny et al., 2005). Interactive motivational counselling approaches, involving discussions and demonstrations of optimal behaviours, can enhance self‐efficacy and subsequent behaviour change (Bergeron et al., 2017). Child growth outcomes, cognitive development and health, as posited by the social–ecological model, are functions of children's genetic makeup, the family and the community in which they live (Bronfenbrenner, 1994). Thus, effective BCCs should consider the family and community contexts. An often‐used model in BCC is based on social cognitive theory (SCT; Bandura, 1986; Perry, Baranowski, & Parcel, 1990), in which environmental influences, personal factors and attributes of the behaviour itself may influence behaviour change. For sustained behaviour change to occur, an individual must believe in his or her capability to perform the behaviour and must perceive that the behaviour is beneficial or rewarding and that the outcome is of sufficient value to motivate the continued performance of the specific behaviour. Encouraging and showing caregivers how to practice nutrition and care for development messages, and delivering these messages through suitable communication channels, facilitate the development of positive behaviours for individual, community and societal behaviour change (United Nations Children's Fund [UNICEF] Regional Office for South Asia, 2005).

On the basis of the principles of BCC, we developed and tested a cluster randomized intervention trial in Andhra Pradesh (now Telangana State), India, to improve infant and young child feeding and care for early child development. The overall aim of the trial was to determine whether an educational intervention delivered to caregivers, which focused on responsive feeding and mother/child interaction, in addition to messages about appropriate breastfeeding and complementary feeding from 3 to 15 months of age, would improve adequacy of dietary intake, iron status, growth and early child development. Details of the broader objectives of this randomized controlled trial and the primary outcomes related to growth and nutrition status were reported in an earlier publication (Vazir et al., 2013).

This paper focuses on the development and use of BCC to examine whether providing caregivers with knowledge and skills, delivered through interactive sessions focusing on responsive parenting interactions during feeding and play, would result in improvements in caregiving quality and improved mental and psychomotor development of young children at 15 months of age.

## 2 METHODS

### 2.1 Sample size, participants and study design

We recruited and enrolled a sample of 600 mother/infant pairs from 60 villages in rural South India. The villages were matched on population size and maternal literacy. The sample size was calculated (Jekel, Elmore, & Katz, 2001) to detect a mean difference in the primary outcome variable of 100 kcals (SD = 265 kcal) in food intake by the infants and accounting for the clustering effect (between groups variance = 0.21) and loss to follow‐up of 20%. This resulted in a required sample size of 600 (α = 0.05 and 80% power) enabling analysis of all the outcome variables including child development (a difference in Bayley's mean score = 3.8, SD = 10). Local women who were mothers (one from each village) with a minimum high school level education were selected and trained as village‐level workers (VW). These women served as peer counsellors to the women who enrolled in the study. The enrolment period continued for 6 months to achieve the required sample of 600 pregnant women from 60 villages. In this setting, pregnant women typically go to their natal home for delivery and then return to their husband's home when the infant is 3 months old. The VW identified pregnant women in their third trimester of pregnancy (preparing to leave for their natal home), and project staff explained the study objectives and obtained informed consent.The Institution Ethics Committees of the National Institute of Nutrition, Indian Council of Medical Research, India, and the University of North Carolina Institutional Review Board, Chapel Hill, USA, approved the study.

A cluster randomization design was the design of choice so as to limit the likelihood of contamination. Sixty villages were selected purposively from three Integrated Child Development Services (ICDS) project areas—the largest multiservices government programme for maternal and child nutrition, health and development in India. Sets of three villages that matched on population size, maternal literacy and birth rate using the then‐latest census figures (2001) were grouped to form strata to allow a stratified random allocation of village clusters across the three arms of the study until there were 20 villages per group. This resulted in a total of 60 villages that provided the required sample of 600 pregnant women.

### 2.2 Study groups

#### 2.2.1 The control group (C group)

Mothers and infants in this group received the standard of care provided by the ICDS programme, which is implemented across the country by the government and functioned across all study groups. These services are mainly centre based. ICDS provides a supplementary meal every day for 6 days a week to the beneficiaries, including pregnant and nursing mothers (from pregnancy to 6 months of lactation). It provides infants and toddlers (6 to 24 months of age) take‐home rations and for 3‐ to 5‐year‐olds a centre‐based meal. Home visits to provide counselling on breastfeeding and complementary feeding, monthly growth monitoring and nonformal preschool education are also provided by the ICDS.

#### 2.2.2 The complementary feeding group (CF group)

Besides the ICDS services, mothers in this group received 11 nutrition education messages from the VW through 30 home visits during the entire intervention period on breastfeeding and complementary feeding. At each home visit, an age‐appropriate nutrition message was delivered to the caregiver using flip charts, demonstrations and counselling sessions.

#### 2.2.3 The responsive complementary feeding and play group (RCF&P group)

Mothers in this group received the ICDS services, plus messages on complementary feeding as in the CF group. Additionally, they received eight messages on responsive feeding and eight developmental stimulation messages, which employed five simple age‐appropriate toys to demonstrate and engage mothers in interactive play during the visit. After the session, the toy was left in the household to encourage interactive play. At each home visit, the VWs delivered the age‐appropriate messages, beginning with the responsive feeding message followed by the play message.

### 2.3 Formative research

We based the ideal behaviours for the intervention on the PAHO (2003) guidelines for IYCF. However, we first conducted formative research to develop the BCC material. The formative research explored important concepts for the development of the intervention including (1) attitudes and perceived social norms related to breastfeeding, (2) beliefs about introducing complementary foods and the first foods fed to the baby, (3) knowledge of infant cues of hunger and satiety and attitudes towards responsive feeding techniques, (4) practices of safe food preparation and (5) the quality and adequacy of foods available within the household. For early child development, the following topics were explored: (1) caregivers' awareness of developmental processes and milestones, including gross motor and fine motor development; (2) language (sounds, words, receptive and expressive language and verbal interaction); and (3) infant emotional states and cues. We also included the household availability of toys for play and the extent to which mothers provided the child with toys to play with. Physical characteristics of the environment including both the household and community, cultural and economic, were also studied as these environmental components influence intervention development and delivery (Bentley et al., 2014). The methods for the formative research included in‐depth interviews, focus group discussions and video‐recorded observations of feeding and play. The data were triangulated and analysed. Through this formative research, the knowledge and beliefs, motivations and aspirations of mothers/families, infant foods and feeding practices, number of meals, quantity of food offered, styles of feeding, child stimulation and local terms and their usage were determined. The local constraints and opportunities for changing behaviours related to IYCF and child development were identified.

The triangulated data were fit into a matrix that had the desired behaviour or the ideal behaviour contrasted with the behaviour that was practised in the community. The matrix included the barriers identified that prevented the practice of the ideal behaviour. Similarly, opportunities for change were also identified that were used to develop the BCC. The aspirations and motivations expressed by the caregivers and the community were built into the BCC content to promote the ideal behaviour.

For example, during formative research, caregivers were asked what they would do when they gave a toy to the child. Most caregivers said they would show the child how the toy worked. The videos of the play session corroborated this, and these demonstrations were often done in an imperative style by the caregiver. Examples of barriers included when some mothers said that ‘they [children] are too young to know how to play with some toys’, necessitating the need to ‘show the child how to play with a new toy’. Others said they were ‘too busy and just leave the toy with the child’ and ‘he/she eventually figures out how to play with the toy’, or ‘smart children figure out for themselves how the toy works.’ We used this as an opportunity to reframe play by emphasizing that the child will experience joy and learning from exploration, resulting in the message, ‘Give the child time to explore.’

### 2.4 Development of the BCC material

The constructs of SCT (Bandura, 2004) were used to facilitate the adoption of the recommended behaviours. The tenets of SCT that were adopted included (i) behavioural capability, i.e. the ability to perform a behaviour through providing essential knowledge and skills, (ii) observational learning of behaviours conducted by others, (iii) reinforcement of behaviours to facilitate continued adoption and promoting the expectation that consequences of their actions will result in benefit and (iv) enhancing self‐efficacy to perform the behaviour.

Based on SCT, messages were developed that would motivate mothers to adopt the recommended behaviours. Motivations focused on immediate benefits (e.g., your child will be happy and be less fussy if she/he is fed at least three meals a day) or long‐term benefits (e.g., playing with your child can make your child smart and he/she will do well in school).

### 2.5 Construction of the messages for counselling at the home visits

For each of the recommended behaviour, the rationale for adopting this behaviour was explained, the action demonstrated, understanding checked and motivation given. Examples of each step for developing complementary feeding and stimulation through play messages follow.

#### Complementary feeding message:

Behaviour *Give your baby food of soft thick consistency from six months.*

Rationale ‘Mother's milk is best for the baby until six months old. But when your baby is six months, mother's milk is not enough as your baby is growing fast. From this age, he/she is ready to eat other foods and can digest thick soft food, thick foods give your baby more energy and nourish him/her more than thin foods. Thick foods are tastier and your baby is ready to swallow and digest these foods at six months.’

Action demonstrated The explanation was followed by showing a simple recipe to make a soft, thick food for the baby.

Checking questions were then added to check understanding and potential action: ‘Tell me how you will prepare the food you will give baby from six months? How confident do you feel about your child eating thick food and eating this recipe of milk and rice?’

Lastly, motivation was provided to encourage the caregiver: ‘You will see how your child likes this thick soft food and how he/she will be more satisfied.’

At the follow up home visits, the understanding, implementation and motivations for the recommended behaviour were reviewed and reinforced, difficulties and possible solutions discussed and encouragement to continue given.

#### Stimulation through play messages:

Behaviour *Give the child time to explore.*

Rationale ‘Your baby will explore and observe everything around him/her so give opportunities to do so. Young children explore with their mouths, so make sure that objects are clean before giving them to the child. Even when the child is given something like a toy or an article you can first allow him/her to explore and observe it on his/her own to see what he does with it! By carrying the child upright you can help him/her have a better view of his/her surroundings; he/she will observe and learn more.’

Action demonstrated The message was followed by giving the mother a toy (rattle) to give to the child and with the instruction to let the child explore it without intervention or assistance.

A checking question asked ‘What will you do when you first give your child a toy/object/snack? How can you help your baby explore objects? What must you be sure of before giving your child the object?’

Motivation The motivation associated with this message was ‘*As infants like to explore with eyes, mouth, and hands, by giving them opportunities to explore you are helping them learn and develop their intelligence*’. Demonstrations can be provided once the infant had the time to explore on his/her own. The use of terms such as smart, clever, intelligence and doing well at school were used as motivations for the play messages, based on caregiver aspirations for their child to be smart and intelligent and to do well in school.

All the messages were pretested to ensure understanding, feasibility for adoption and cultural acceptability. Photographs modelling specific behaviours were produced and were incorporated into the age‐specific flip charts. The key intervention messages are given in Box 1.

**BOX 1:** Messages in the intervention flip chart used by village workers to deliver the intervention to mothers/caregivers

Complementary feeding messages (*n* = 11)	Responsive feeding messages (*n* = 8)	Developmental stimulation messages (*n* = 8)
Continue frequent on demand breastfeeding	Smile and respond to your baby during feeding	Smile & look into your child's eyes
Give food of soft, thick consistency.	Teach your child to eat patiently and lovingly	Respond to child's sounds, gestures and interests
Give pulses daily	Actively help your child to eat	Give time to explore objects/persons/things
Give animal foods: egg, milk, meat, liver when you eat them	Respond to cues of hunger, satiety and rejection	Help your child find new things to do with familiar toys
Give X (age appropriate amount) of food at each meal	Help, but do not physically restrain the child	Play simple games, e.g., peek‐a‐boo
Give dark green/orange vegetables/fruits	Praise, encourage child to eat; give positive comments	Talk to child and give names for things and people
Give food three times a day	Response to child refusal is to offer one more bite	Ask your child simple questions
Continue feeding during illness; increase during convalescence	Encourage self‐feeding and give child chewable ‘finger‐foods’ to eat	Explain things and show children how to do things
Give spoonful of oil or ghee		
Give ICDS fortified food		
Wash your & child's hands before preparing food & feeding		

The CF Group received 11 messages on complementary feeding only. The RCF&P Group received all messages given in the box. Messages delivered were dependent on the age of the child. Source: Vazir et al., 2013.

**FIGURE 1:**
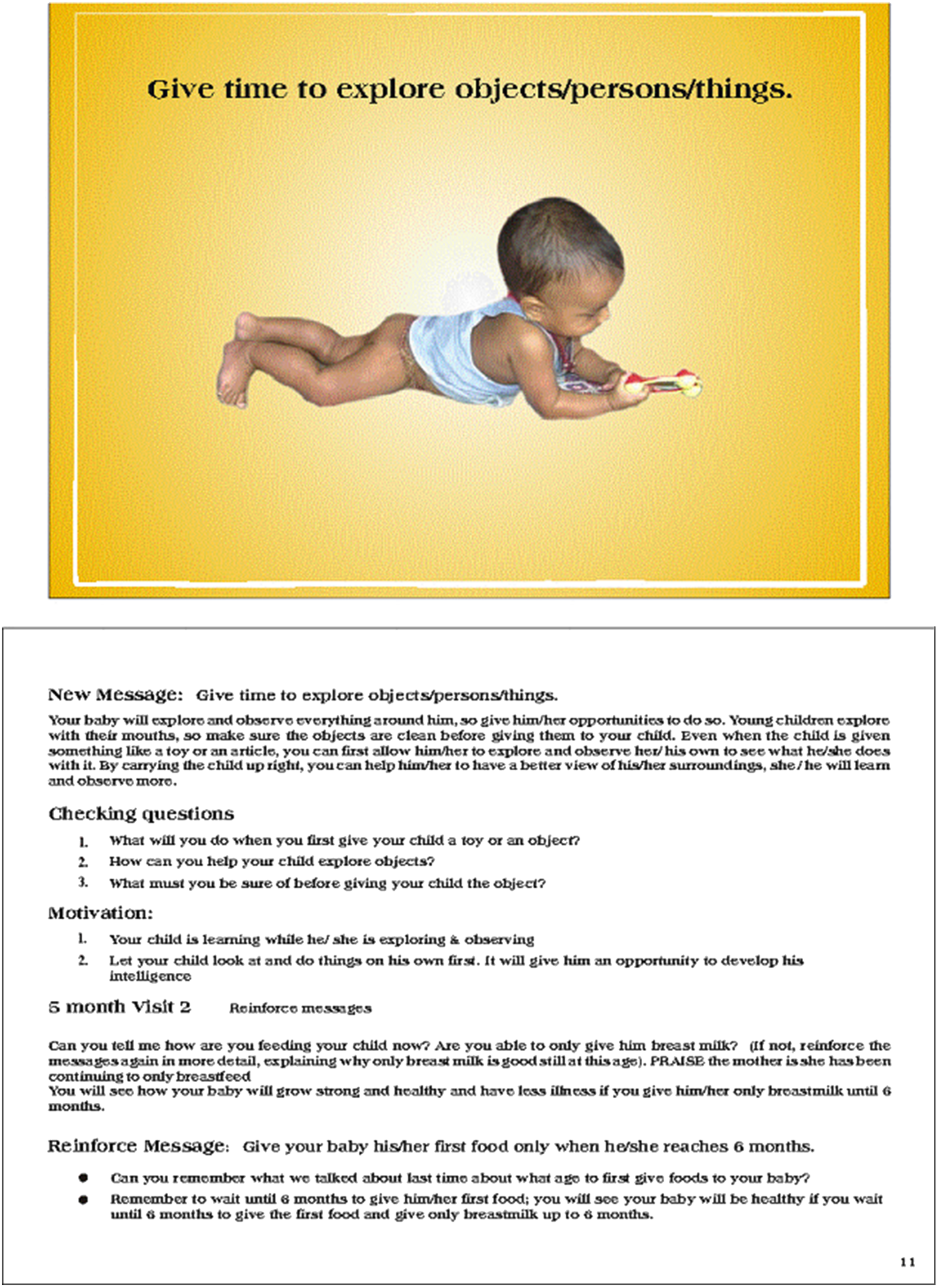
Example of a message displayed, with illustration, in the flip chart along with reinforcers, knowledge and motivations to be delivered and checking questions to ensure reception of message

### 2.6 Training and intervention delivery

Training sessions for VWs, led by project supervisors, were organized in groups and provided separately for the two intervention arms. During the week‐long trainings, the supervisors instructed VWs on how to use a pictorial flip chart and to have ‘focused conversations’ with mothers for the various intervention topics. They demonstrated rapport building and listening skills through role play and encouraged the VWs to praise the mother when she showed positive behaviours during the home visit. As messages were delivered according to the child's age, in‐service training was also provided once every 3 months, addressing the messages relevant to that age interval. The in‐service training also ensured the quality of the intervention was maintained throughout the study period.

Home visits were scheduled at a time that was convenient for the mother and when the child was likely to be awake. For the RCF&P group, the VWs demonstrated and engaged mothers in how to use the toys for interactive play during the visit. Both intervention arms were scheduled to receive the same number of home visits from the VW, which were (1) biweekly when the infant was 4 to 6 months old; (2) weekly visits from thereon, until the infant was 9 months (as this was deemed a critical period of transition from breastfeeding to complementary feeding) and, thereafter, (3) biweekly visits until the toddler was 14 months old (Vazir et al., 2013). Thus, the duration of the intervention spanned 1 year. Every home visit made by the VW was recorded in a monitoring form, which included date and time of the visit, name and the number of the message delivered, caregiver's responses to checking questions, troubleshooting addressed and date of the next visit. The project supervisor checked the monitoring forms weekly.

### 2.7 Assessment timing and procedures

A battery of assessments was performed over the duration of the study to measure each outcome of the intervention. Baseline assessments of infants and mothers were conducted when the infants were 3 months of age, including anthropometry, haemoglobin status, birth history, socio‐demography and socio‐economic status. The final assessment was performed when the children reached 15 months of age. Child development was assessed only at 15 months.

The Home Observation for Measurement of the Environment (HOME) scale, a commonly used and validated instrument, was implemented to assess the home environment (Bradley & Corwyn, 2005; Bradley, Corwyn, Burchinal, McAdoo, & García Coll, 2001; Grantham‐Mcgregor et al., 2007; Hamadani, Fuchs, Osendarp, Huda, & Grantham‐McGregor, 2002). The scale has six subscales, which include (1) Emotional and Verbal Responsivity of Caregiver, i.e. the degree to which caregivers respond to the infant (emotional, physical and communication); (2) Avoidance of Restriction and Punishment, i.e. the child has opportunities to learn through trial and error and the child is not punished; (3) Caregiver Promotes Child Development, i.e. opportunities for the child to engage in developmentally appropriate and scaffolding activities; (4) Organization of Physical and Temporal Environment, i.e. the child's physical environment is organized; (5) Provision of Play Materials, i.e. the infant is provided material to develop competence and for enjoyment and exploration, and (6) Opportunities for Variety in Daily Stimulation, i.e. the child is provided opportunities for a variety of experiences. In the present study, we administered the HOME scale at 3, 6, 9, 12 and 15 months of infant age. We also included cleanliness questions, such as ‘is the infant kept clean (bathed), no offensive odour, clothes are clean’, because during formative research, we found the cleanliness of the infant to be highly variable among households.

The Bayley Scales of Infant Development‐II were used to assess the mental and psychomotor development indices (MDI and PDI). Raw scores obtained were transformed into index scores based on the norms (Bayley, 1993). The assessment was performed only at 15 months at home in the caregiver's presence.

Because symptoms of maternal depression have been associated with poorer child growth and development ( Liu et al., 2017; Rahman, Iqbal, Bunn, Lovel, & Harrington, 2004), maternal depression symptoms were assessed using the Centre for Epidemiological Survey Depression scale (Radloff, 1977) at baseline and 6, 9, 12 and 15 months post‐partum (data not presented). In this paper, as maternal depression was employed as a covariate, only baseline data are presented.

Assessments for infant development and the home environment were conducted by a team of psychologists trained by one of the senior investigators of the study. The interrater reliability was determined to be 94%–98%. Assessors also received periodic training throughout the study to maintain quality and reliability and prevent drift. The psychologists doing the assessments were blinded to the intervention provided to the participants.

### 2.8 Statistical analyses

Data analyses were performed with SPSS, version 19.0 (SPSS Inc., Chicago, IL, USA.) The first step in the analyses compared the intervention groups at baseline adjusting for clustering effects using descriptive statistics. Analyses of baseline data assessed differences using descriptive statistics among the three groups in socio‐demographic factors, maternal factors (height, weight and symptoms of depression) and measures of anthropometry and infant feeding. All results reported were adjusted for cluster randomization using mixed models for continuous variables. Estimates for categorical variables were produced using generalized estimating equations. Mixed model analyses focussed on differences between intervention groups in the HOME score and the Bayley's derived mental and motor scores at 15 months of age as well as the association between HOME score and development. Regression analyses were performed to estimate the percentage of variance in development (Bayley's mental and motor index) explained by subscales of the HOME score. We also assessed whether controlling for any of the baseline characteristics of infants, mothers or households changed these relationships. The significance level used for statistical testing was *P* < 0.05.

## 3 RESULTS

The intervention was delivered for 12 months. At baseline, there were no differences among the three groups in any of the variables tested. Table 1 provides details on infant and maternal characteristics. The mean birth weight of the infants was 2.82 ± 0.43 kg. At 6 months, nearly all (99.6%) the infants were being breastfed. The mean maternal age was 22.2 years, and 38.6% of the women were first‐time mothers. There were no differences among groups in maternal education and family economic status. Maternal depression symptoms and HOME score also showed no difference at baseline.

**TABLE 1:** Baseline characteristics of infants and mothers in the three study groups

Variable	Group		
	C group *n* = 199	CF group *n* = 207	RCF&P group (*n* = 194)
Males	50.8	48.3	49.0
Mean birth weight (kg)	2.8 ± 0.5	2.9 ± 0.5	2.8 ± 0.5
Term births (%)	84.4	88.3	86.6
Weight at 3 months (kg)	5.5 ± 0.8	5.6 ± 0.9	5.6 ± 0.7
Length at 3 months (cm)	58.9 ± 2.5	59.0 ± 2.6	59.4 ± 2.4
Mean child haemoglobin (g dL^−1^) at 3 months (*n* = 578)	9.0 ± 0.6	8.9 ± 0.7	9.0 ± 0.8
Breastfed at 6 months (%)	99.4	99.5	100
Primigravida	40.7	40.3	34.7
Mean maternal age (years) (*n* = 596)	21.9 ± 3.1	22.3 ± 3.5	22.3 ± 3.4
Maternal education (%) Illiterate Secondary or high school education	37.4 27.2	38.9 25.3	38.2 32.0
Maternal depression score (*n* = 589; mean ± SD)	30.8 ± 8.5	30.4 ± 8.1	30.1 ± 8.3
HOME score (*n* = 414; mean ± SD)	80.1 ± 3.4	80.7 ± 3.3	80.8 ± 3.8

*Note*: Variables did not differ significantly (*P* < 0.05) across groups. Data presented as mean and percentages using cluster adjusted analysis of variance with mixed models and generalized estimating equations.

Abbreviations: C group, Control group; CF group, complementary feeding group, RCF&P group, responsive feeding and play group.

**TABLE 2:** Mean and SD of HOME score and Bayley Mental & Motor Development for each study intervention group at 15 months

Group	Total HOME score	Mental development	Motor development
C group (*n* = 182)	87.2 ± 3.4^a^	104.4 ± 8.6^a^	114.3 ± 14.3
CF group (*n* = 176)	88.2 ± 2.9^b^	105.6 ± 8.4^a, b^	115.7 ± 14.1
RCF&P group (*n* = 153)	88.3 ± 2.5^b^	107.4 ± 8.1^b^	116.7.4 ± 14.2

*Note*: Data present mean and standard deviation adjusted for standard of living index, birth weight, maternal height, maternal depression score at 3 months, maternal education and scheduled caste/tribe community in the regression equation. Means in a column with superscripts without a common letter differ, *P* < 0.05 (cluster‐adjusted ANCOVA with mixed model). The C group (a) differs from both CF group (b) and RCF&P group (b) on HOME score. The C group (a) differs from RCF&P group (b) but not from CF group (a,b) in mental development. The CF group (a,b) and RCF&P group (b) show no significant difference in mental development.

The mean number of visits received by the two intervention groups was 29.5 ± 9.3 for the CF group and 25.7 ± 9.6 for the RCF&P group, and the difference was significant at *P* = 0.001. The mean duration of a visit was 17.8 ± 5.4 min for the CF group and 26.5 ± 7.0 min for the RCF&P group, and the difference in minutes was significant at *P* = 0.001.

The total HOME scores did not differ at baseline or at the next three time points (data not shown). At 15 months, it differed by group, *F*(2, 38) = 6.41, *P* < 0.001, with the CF and RCF&P groups scoring significantly higher than the C group (*P* < 0.05) (Table 2).

The MDI scores differed by intervention group, *F*(2, 37) = 3.31, *P* = 0.04 (Table 2), at 15 months. Scores were significantly higher in the RCF&P group than in the C group (*P* = 0.02) but not in the CF group (*P* = 0.19). The difference between the C and RCF&P groups continued to be significant when controlling for socio‐economic status and other potentially confounding variables including birth weight, maternal height, maternal education, household assets and maternal depression symptoms at baseline. We found no statistically significant differences for the PDI among groups although scores for this measure were lowest in the C group.

**FIGURE 2:**
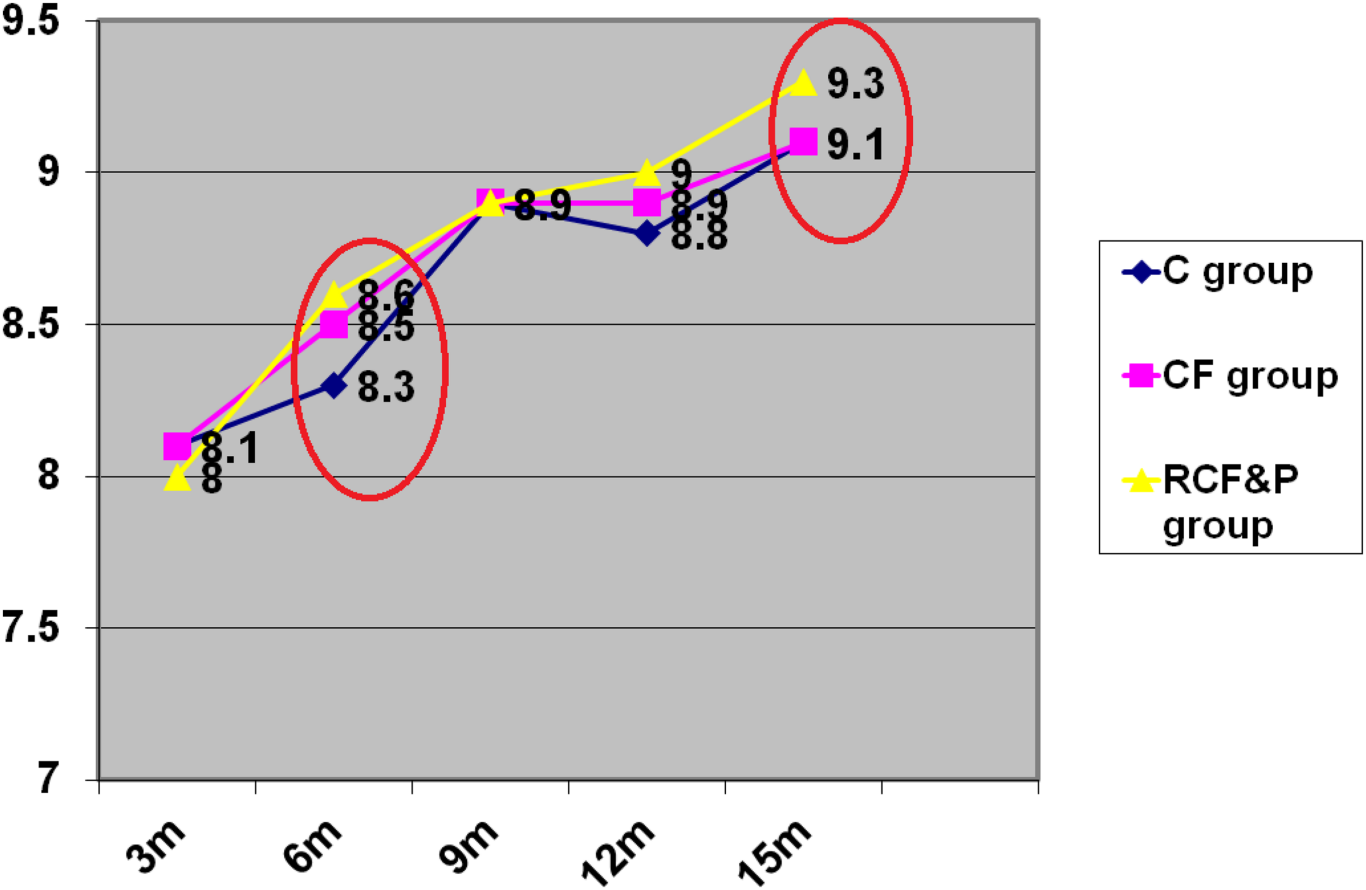
HOME subscale: Caregiver promotes child development mean and SD at five time points in the three intervention groups (*n* = 521). Note: C group, control group; CF group, complementary feeding group; RF&P group, responsive feeding and play group. Data presented are mean and standard deviation adjusted for maternal education and standard of living index. CF and RCF&P groups > C group at 9 and 12 months. RF&P group differed from C and CF groups at 15 months, *F*(2, 519) = 8.14, *P* = 0.000

When examined by subscale, few differences in the HOME were noted at any time point, with two exceptions. At 15 months, the RCF&P group scored higher than the C group and the CF group on two subscales: ‘Caregiver Promotion of Child Development’, *F*(2, 519) = 4.96, *P* = 0.007 (Figure 2), and ‘Opportunities for Variety in Daily Stimulation’, *F*(2, 519) = 8.14, *P* = 000. Controlling for maternal education, depression and socio‐economic status did not change the pattern of results.

HOME subscales scores at 15 months that were associated with MDI and PDI were ‘Caregiver Promotes Child Development’ (*F* = 25.38; *P* < 0.001) and ‘Cleanliness of the child’ (*F* = 6.33;*P* = 0.012) (Table 3). The variance explained for MDI by these two subscales was 7.8%. The subscale ‘Caregiver Promotes Child Development’ alone was associated (*F* = 15.96; *P* < 0.001) with PDI, explaining 3.9% of the variance in PDI.

**TABLE 3:** Subscales of the HOME associated with mental (MDI) and motor development index (PDI) in children in the three study groups at 15 months^a^

Subscales *n* = 501	Unstandardized coefficients with 95% CI	*P* value	Unstandardized coefficients with 95% CI	*P* value
Emotional and verbal responsivity of caregiver	0.136 [−0.625, 0.896]	0.726	−0.107 [−1.392, 1.178]	0.870
Avoidance of restriction and punishment	−0.417 [−2.181, 1.346]	0.642	−0.087 [−3.066, 2.893]	0.954
Caregiver promotes child development	2.433 [1.361, 3.504]	0.000	3.254 [1.444, 5.065]	0.000
Organization of physical and temporal environment	−0.008 [−0.620, 0.605]	0.980	−0.236 [−1.271, 0.799]	0.655
Provision of appropriate play materials	0.567 [−0.412, 1.545]	0.256	1.092 [−0.561, 2.746]	0.195
Opportunities for variety in daily stimulation	0.416 [−0.236, 1.067]	0.211	−0.173 [−1.274, 0.928]	0.757
Cleanliness of child	−3.117 [−5.695, 0.538]	0.018	−2.330 [−6.686, 2.027]	0.294
*R* ^2^ (%)	9.0		6.8	
Significant variables Contribution *R* ^2^ (%)	7.6		3.9	

*Note*: Cluster‐adjusted analysis of variance with mixed model was fitted for mental and motor development with standard of living index, birth weight, maternal height, maternal education and maternal depression at 3 months, and infants age as covariates and subscales of the HOME as predictors (*P* < 0.05). Regression analysis was carried out for MDI and PDI with subscales of HOME at 15 months.

^a^ Dependent variable mental development index and motor development index.

## 4 DISCUSSION

This efficacy trial tested an educational intervention of complementary feeding, responsive feeding and play for mothers/caregivers of 3‐month‐old infants. The duration of the intervention was 1 year, and outcomes tested were the home environment and early child development. The BCC content used in the study was developed through formative research and based on the socio‐ecological and social cognitive theories (Bandura, 1986; Bronfenbrenner, 1994). There were no baselines differences among the three arms of the study. The monitoring data of our trial suggest that although both intervention groups were to receive the same number of visits from the VW, the CF group received visits closer to the actual scheduled number of 30 visits compared with RCF&P group. A mother and her infant not being at home was the most common reason for a missing home visit, and at least two follow‐up visits were made by VWs if the caregiver was not present during the first visit. The HOME scale was administered at five time points, and a significant difference among groups was noted only at the endpoint of 15 months. Our study confirms the findings of other early child development interventions that have improved the total HOME score following its completion (Prado et al., 2017; Walker, Chang, Powell, & Grantham‐McGregor, 2004; Yousafzai, Rasheed, Rizv, Armstrong, & Bhutta, 2015). In the present study, both the CF and RCF&P groups had higher HOME score than the C group. The RCF&P group did not differ from the CF group in the total HOME score, despite receiving detailed responsive feeding and play stimulation intervention content. Our results contrast with a number of studies in which large effects were reported for mother–child interaction and the caregiving environment when responsive care and stimulation was focal to the intervention (Nahar et al., 2014; Orri, Côté, Tremblay, & Doyle, 2019; Tessier et al., 2009; Yousafzai et al., 2015). We, however, report significant difference on two subscales of the HOME scale: ‘Opportunities for Daily Stimulation’ and ‘Caregiver Promotes Child Development’ in the RCF&P group, compared with the C and CF groups from 9 to 15 months. These scales were closely aligned with the messages that were being delivered to the RCF&P group in the intervention and may be associated with the difference among groups in these two subscales at 15 months.

The intervention also resulted in a higher mental index score at 15 months, but not psychomotor index score, in the RCF&P group, which received the intervention to promote cognitive development through stimulation and play compared with the C group. Responsive care and stimulation interventions have often improved children's cognitive abilities and prosocial behaviours compared with routine services (Aboud & Yousafzai, 2015; Grantham‐McGregor & Walker, 2015; Smith, Landry, & Swank, 2000; Tucker‐Drob & Harden, 2012; Worku et al., 2018), whereas early nutrition interventions have shown a smaller effect on children's cognitive development in low‐ and middle‐income countries (Grantham‐McGregor, Fernald, Kagawa, & Walker, 2014; Larson & Yousafzai, 2015). In this study, there were no improvements noted in motor development in either of the intervention groups when compared with the control group. Hartinger et al. (2017) reported significant improvement across all domains of development and attributed these improvements, at least in part, to the strategy of engaging qualified professional home visitors to deliver the intervention. Fine motor domains are likely to require intensified interaction between the child and the caregiver, which was not explicitly promoted in our intervention. Our intervention deliverers did receive basic training but were not professionally qualified, which might explain the differences in our findings.

The duration of visits for the RCF&P group was longer (~25 min) that for the CF group (~18 min). However, interactive play interventions have employed longer home visits (60–90 min) than the present study and have reported positive outcomes as a result of these longer visits (Martinez et al., 2018). In a low‐ or middle‐income country context such as India, the cost of longer visits has to be considered within the programming strategy and weighed against the benefits that it achieves when resources are limited. Our findings suggest that although the RCF&P group intervention was more costly to deliver, this extra investment is associated with incremental gains in mental development of an average of 3 units. The magnitude of benefit, although somewhat less than the increase associated with clinical significance of 5 units (Vohr et al., 2006), could be considered important at a population level.

We also examined the association of the subscales of HOME with infant mental and psychomotor development. The relationship between the HOME, its subscales and cognitive development has been well documented (Andrade et al., 2005; Jones et al., 2017; Ranjitkar et al., 2019). In this study, though not high, the variance explained by two subscales is sufficient to establish a significant association. Caregiving that incorporates play and learning into daily activities in the home has been associated with improvements in cognition (Prado et al., 2017; Victora, Victora, & Barros, 1990), with benefits into adulthood (Walker, Chang, Powell, & Grantam‐McGregor, 2005). The cleanliness subscale associated with cognitive development at 15 months may demonstrate the importance of hygiene in development. Our intervention included only one message on hygiene ‘Wash your & child's hands before preparing food & feeding’.

### 4.1 Lessons learnt

The lessons learnt from this study is that the time spent in the home visit for the RCF&P group may have been too short to have sufficiently relayed the number of messages and instructions for play delivered in one visit. Research focusing on strategies to help mothers incorporate messages into their daily activities (without overburdening them) is recommended.

## 5 CONCLUSION

This integrated intervention of nutrition and child development provided support for infant feeding and child development through play. There is a need for programmes to support caregivers in how to provide nurturing care and for the appropriate training of health workers and nonspecialists (Britto et al., 2017). Early child development interventions may be most effective when they are comprehensive, of high quality, of sufficiently long duration and appropriately timed to provide such information during specific developmental stages of the infant (Engle et al., 2007; Shonkoff, 2010). This integrated intervention included these important components and did result in some improvement in home environment outcomes and infants' mental development at 15 months compared with the groups who did not receive this intervention content.

## CONFLICTS OF INTEREST

All authors (Fernandez‐Rao, Bentley, Griffiths, Balakrishna, Creed‐Kanashiro and Johnson) declare ‘no conflict of interest’.

## CONTRIBUTIONS

SFR, PE, SV, MEB, SLJ, HC‐K and PG conceptualized and designed the research presented in this paper. SV and SFR conducted the research; NB and PG analysed the data; SFR, SLJ, SV, MEB, HC‐K, PG and NB, interpreted the data; SFR drafted the paper; SFR, SLJ, MEB, PG, NB and HC‐K had primary responsibility for review and editing of the final content. All authors read and approved the final manuscript.
